# Genome Sequence of *Bacillus velezensis* S141, a New Strain of Plant Growth-Promoting Rhizobacterium Isolated from Soybean Rhizosphere

**DOI:** 10.1128/genomeA.01312-17

**Published:** 2017-11-30

**Authors:** Surachat Sibponkrung, Takahiko Kondo, Kosei Tanaka, Panlada Tittabutr, Nantakorn Boonkerd, Neung Teaumroong, Ken-ichi Yoshida

**Affiliations:** a School of Biotechnology, Institute of Agricultural Technology, Suranaree University of Technology, Nakhon Ratchasima, Thailand; b Graduate School of Science, Technology and Innovation, Kobe University, Kobe, Japan; c Organization of Advanced Science and Technology, Kobe University, Kobe, Japan

## Abstract

*Bacillus velezensis* strain S141 is a plant growth-promoting rhizobacterium isolated from soybean (*Glycine max*) rhizosphere that enhances soybean growth, nodulation, and N_2_ fixation efficiency by coinoculation with *Bradyrhizobium diazoefficiens* USDA110. The S141 genome was identified to comprise a 3,974,582-bp-long circular DNA sequence encoding at least 3,817 proteins.

## GENOME ANNOUNCEMENT

Plant growth-promoting rhizobacteria (PGPR) are naturally occurring soil bacteria that aggressively colonize plant roots for better plant growth (1). Various bacterial species have been reported as PGPR, including some *Bacillus* species (2). Several *Bacillus* strains currently promote plant growth through the suppression of disease and improvement in nutrient acquisition by functioning as biofertilizers or phytohormone producers (1, 3). *Bacillus velezensis* S141 is a PGPR isolated from soybean rhizosphere soil in Thailand and is closely related to *B. subtilis* GB03 based on 16S rRNA gene sequencing (4). *B. velezensis* S141 is capable of increasing soybean growth, nodulation, and N_2_ fixation efficiency via coinoculation with *Bradyrhizobium diazoefficiens* USDA110. Therefore, we aimed to determine the whole-genome sequence of *B. velezensis* S141 to reveal the genetic background of its soybean growth-promoting capacity involving symbiotic N_2_ fixation with *B. diazoefficiens* USDA110.

*B. velezensis* S141 genomic DNA was prepared using the Wizard Genomic DNA purification kit (Promega, USA), and a genomic DNA library was constructed using the NEBNext ultra DNA library prep kit for Illumina (New England BioLabs Inc., USA) according to the manufacturer’s protocol. *B. velezensis* S141 genome sequencing was performed on an Illumina MiSeq platform. After quality filtering, 11,675,054 high-quality paired-end reads (2 × 300 bp) were assembled using CLC Genomics Workbench version 10.0.1 (Qiagen, USA). Total read coverage was 300-fold. After trimmed (paired) assembly using the CLC Genomics Workbench, a draft genome with 19 scaffolds was obtained. Gaps between scaffolds were closed using PCR and Sanger sequencing to obtain the complete sequence of the circular chromosome. The genome sequence was automatically annotated using the Microbial Genome Annotation Pipeline (MiGAP) (5). Based on 16S rRNA gene analysis using BLAST searches, the highest sequence identities (up to 100%) were identified with *B. amyloliquefaciens*, *B. velezensis*, *B. subtilis*, and *B. methylotrophicus*. Average nucleotide identity analysis using the Map Reads to Reference tool indicated that among all related *Bacillus* species, the *B. methylotrophicus* NAU-B3 (*B. amyloliquefaciens* subsp. *plantarum* NAU-B3) genome sequence (6) showed the highest identity (96.44%) to that of strain S141. Strain NAU-B3 was recently reclassified as *B. velezensis* (7), and thus we decided to name strain S141 *B. velezensis* S141.

The *B. velezensis* S141 chromosome was found to be a 3,974,582-bp-long circular DNA without a plasmid. Its genome comprises at least 3,817 protein-coding genes. Compared with another PGPR *Bacillus* strain, *B. velezensis* FZB42 (*B. amyloliquefaciens* subsp. *plantarum* FZB42) (7), strain S141 contains putative genes involved in indole-3-acetic acid (IAA) production (8), including *ipdC* and *dhaS* encoding indole-3-pyruvate decarboxylase and indole-3-acetaldehyde dehydrogenase, respectively, to synthesize IAA from indole-3-pyruvic acid; putative* iaaH* encoding indole-3-acetamide hydrolase to synthesize IAA from indole-3-acetamide; and *yhcX* encoding nitrilase to synthesize IAA from indole-3-acetonitrile. Additionally, we found *ysnE*, which putatively encodes IAA transacetylase involved in the tryptophan-independent IAA biosynthesis pathway. Thus, we suggest that *B. velezensis* S141 possesses multiple genes that are functionally related to auxin production and play key roles in its ability to promote plant growth.

### Accession number(s).

*B. velezensis* S141 genome sequence was deposited in DDBJ/EMBL/GenBank under accession number AP018402.
